# Xenobiotic-induced activation of human aryl hydrocarbon receptor target genes in *Drosophila* is mediated by the epigenetic chromatin modifiers

**DOI:** 10.18632/oncotarget.22173

**Published:** 2017-10-31

**Authors:** Angelina A. Akishina, Julia E. Vorontsova, Roman O. Cherezov, Il’ya B. Mertsalov, Olga G. Zatsepina, Mikhail S. Slezinger, Vladislav M. Panin, Svetlana Petruk, Grigori N. Enikolopov, Alexander Mazo, Olga B. Simonova, Boris A. Kuzin

**Affiliations:** ^1^ Kol’tsov Institute of Developmental Biology, Russian Academy of Sciences, Moscow, Russia; ^2^ Engelhardt Institute of Molecular Biology, Russian Academy of Sciences, Moscow, Russia; ^3^ Department of Biochemistry and Biophysics, Texas A and M University, College Station, TX, USA; ^4^ Department of Biochemistry and Molecular Biology and Kimmel Cancer Center, Thomas Jefferson University, Philadelphia, PA, USA; ^5^ Center for Developmental Genetics, Department of Anesthesiology, Stony Brook University, Stony Brook, NY, USA

**Keywords:** xenobiotic, aryl hydrocarbon receptor, PcG epigenetic complexes, drosophila

## Abstract

Aryl hydrocarbon receptor (AHR) is the key transcription factor that controls animal development and various adaptive processes. The AHR’s target genes are involved in biodegradation of endogenous and exogenous toxins, regulation of immune response, organogenesis, and neurogenesis. Ligand binding is important for the activation of the AHR signaling pathway. Invertebrate AHR homologs are activated by endogenous ligands whereas vertebrate AHR can be activated by both endogenous and exogenous ligands (xenobiotics). Several studies using mammalian cultured cells have demonstrated that transcription of the AHR target genes can be activated by exogenous AHR ligands, but little is known about the effects of AHR in a living organism. Here, we examined the effects of human AHR and its ligands using transgenic *Drosophila* lines with an inducible human *AhR* gene. We found that exogenous AHR ligands can increase as well as decrease the transcription levels of the AHR target genes, including genes that control proliferation, motility, polarization, and programmed cell death. This suggests that AHR activation may affect the expression of gene networks that could be critical for cancer progression and metastasis. Importantly, we found that AHR target genes are also controlled by the enzymes that modify chromatin structure, in particular components of the epigenetic Polycomb Repressive complexes 1 and 2. Since exogenous AHR ligands (alternatively – xenobiotics) and small molecule inhibitors of epigenetic modifiers are often used as pharmaceutical anticancer drugs, our findings may have significant implications in designing new combinations of therapeutic treatments for oncological diseases.

## INTRODUCTION

Many cellular processes in higher multicellular organisms depend on the activity of the Aryl hydrocarbon receptor (AHR); among them are the maintenance of homeostasis, the regulation of detoxification, cell division, differentiation, polarization, programmed cell death, the formation of organ-tissue structures, nervous, immune, cardiovascular, endocrine, generative, and excretory systems [1–14]. AHR is a transcription factor with three functional domains: a highly conserved N-terminal basic-helix-loop-helix (bHLH) domain, a less conserved Per/Arnt/Sim (PAS) domain, and a weakly conserved C-terminal domain [6, 15]. Unliganded human AHR is localized in the cytoplasm where it is associated with the molecular chaperons HSP90 (Heat Shock Protein 90) and XAP2/AIP (X-associated protein 2/AhR-interacting protein). Following binding to the ligand, AHR translocates to the nucleus, dissociates from HSP90, forms a heterodimer with the ARNT (Aryl hydrocarbon receptor nuclear translocator) and binds to specific DNA sequences known as the *Xenobiotic Response Elements*.

In humans and mammals, AHR is activated by a variety of endogenous ligands and xenobiotics (exogenous ligands) [16–19]. While maintenance of a proper concentration of the active (liganded) AHR is important for cell survival and organism functioning [20–24], the changes in AHR expression are rather frequent events. For example, aging is often associated with a decrease in the level of AHR expression. The most dramatic consequences of the decreased AHR expression are an increased risk of cancer and the inability to protect cells against the toxic effects of xenobiotics [25, 26]. Ectopic AHR activation causes a variety of developmental disorders, e.g., abnormal organogenesis and histogenesis, disruptions in the nervous, immune, cardiovascular, endocrine, and generative systems. In humans and vertebrates, the endogenous ligands often function as agonists that enhance AHR activity. There is a wide range of affinities of xenobiotic ligands to AHR [27]. Apparently, the ligand binding affinities can modulate AHR’s ability to activate target genes [28].

Experiments in cultured cells limit the understanding of the effects induced by the AHR expression on the developmental processes in the living organism. To gain a better understanding of the functioning of AHR *in vivo,* we created several ‘humanized’ *Drosophila* transgenic animals, which carry transgenes with the inducible human *AhR* gene under the control of the yeast *UAS* (*Upstream Activation Sequence*) promoter element. These transgenic constructs allow the induction of AHR expression in different organs of *Drosophila* by using various tissue-specific GAL4-drivers [29]. It is believed that in invertebrates, AHR homologs are activated only by endogenous ligands [4, 30]. Therefore, since the majority of xenobiotics activating human AHR are not able to activate the *Drosophila* AHR homolog, this allows the assessment of their specificity of action by introducing them into the *Drosophila* feed medium. Activation of the human AHR in different *Drosophila* tissues and organs allows us to estimate the ability of the human AHR ligands to regulate transcription of the human AHR target genes *in vivo*. It was previously shown that transgenic mouse AHR and *Drosophila* ARNT could form a functional heterodimer capable of inducing dioxin-mediated activation of AHR target gene homologs in *Drosophila* [31]. Here, we demonstrated that AHR activation induced by different exogenous ligands has pleiotropic effects, i.e. it can both increase and decrease transcription of the AHR target genes in different tissues and this effect depends on the developmental stage of the animal. Importantly, we found that AHR’s effect on target genes is mediated by Polycomb group (PcG) epigenetic chromatin regulators. Thus, the results of this study expand our knowledge of the *in vivo* role of the human AHR in the regulation of development and biodegradation of the toxic agents and opens up the possibility of using combinations of xenobiotics and epigenetic inhibitors in the treatment of a variety of diseases.

## RESULTS

### Strong phenotypic effects of endogenous and exogenous human AHR ligands in *Drosophila* tissues

**Figure 1:**
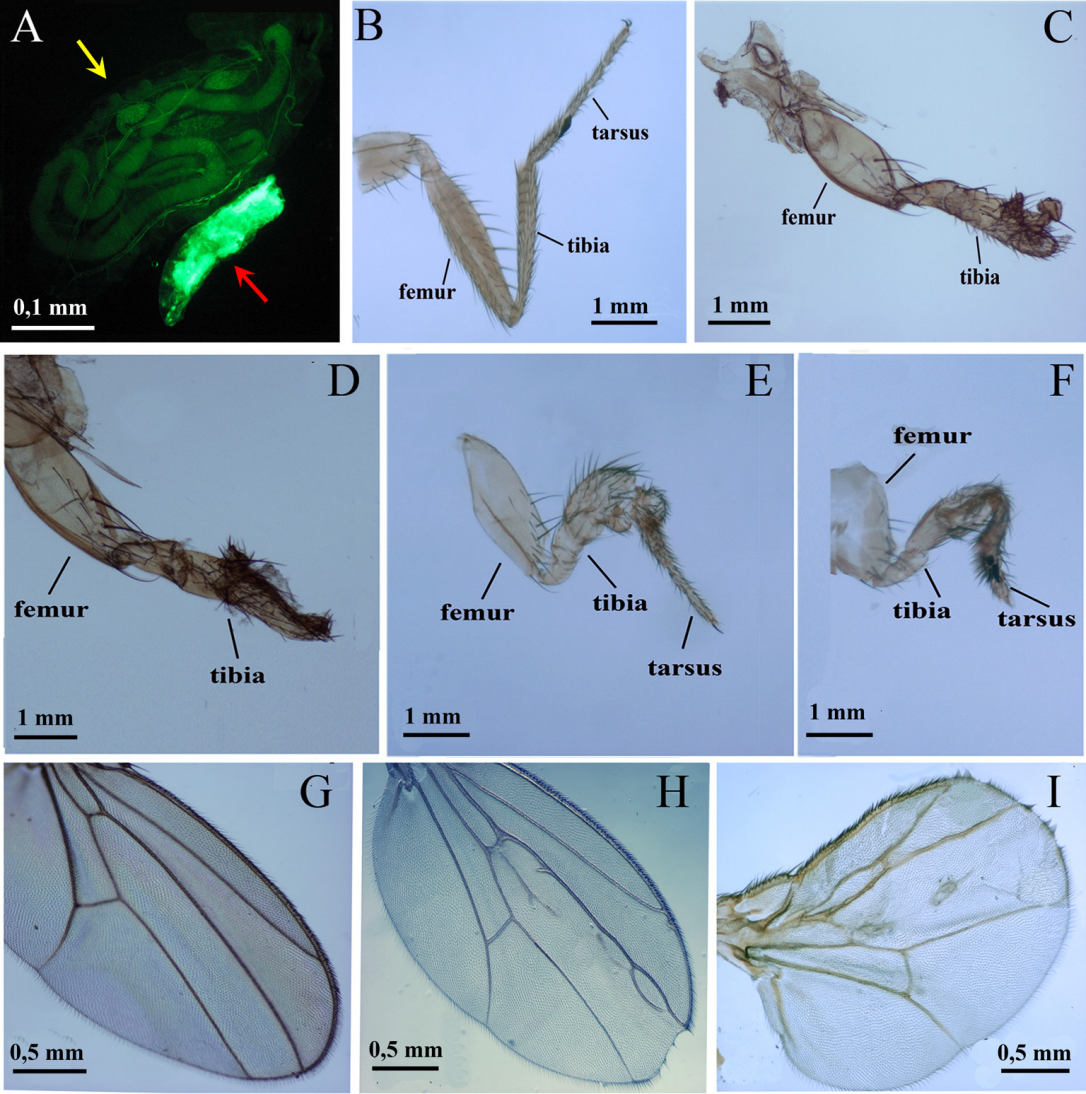
Phenotypic effects of endogenous and exogenous ligands of the human AHR on *Drosophila* growth and morphogenesis (**A**) Ubiquitous expression of *UAS-AhR* leads to developmental lethality. The majority of *tub>AhR* animals die at the embryonic stage, with very few escapers that die at early larval stages, showing arrest in growth and development. Two four-day old larvae are shown, the larger one is the control (*UAS-AhR/+*; *UAS-GFP/+*, yellow arrow), the smaller green larva (red arrow) is *tub>AhR*, with ubiquitous expression of transgenic AHR (*UAS-AhR/+*; *UAS-GFP/Tub-GAL4*). The expression pattern of *tub-GAL4* is visualized by GFP expression (green). (**B**–**C**). *Drosophila* leg phenotypes of *Dll>AhR flies*. (B) control (*UAS-AhR/+*). (C) *Dll>AhR* (*Dll-GAL4/UAS-AhR*). Flies developed on standard medium with no exogenous ligands. (**D**–**I**). *Drosophila* leg (D–F) and wing (G–I) phenotypes of *dpp*>*AhR* flies. (G) control (*UAS-AhR/+*). (D–F, H–I) *dpp>AhR* (*dpp-GAL4/UAS-AhR*). Flies developed on standard medium without exogenous ligands (G–H) or with indirubin (D), indinol (E, I) and beta-Naphthoflavone (F). At least 80 legs, 40 wings and more than 20 flies were analyzed for each genotype. Leg segments are indicated. Note the loss of tarsal segments in (C–D).

It is essential to study the effects of xenobiotics on mammalian AHR *in vivo*. *Drosophila* represents a unique model for these experiments since previous studies have indicated that dioxin and other xenobiotics, which are known to bind to the mammalian AHR, were unable to activate the invertebrate AHR homologue. However, dioxin affected *Drosophila* leg and eye development when the ectopic mouse *UAS-AhR* was induced by the *dpp-GAL4* and *GMR-GAL4* drivers in the primordial leg or eye tissues, respectively [31]. At the same time, it is possible that there are some endogenous ligands that are capable of activating human AHR in other *Drosophila* tissues. To investigate this we used a number of GAL4 driver lines to induce human AHR in different *Drosophila* tissues. Ubiquitous expression of the *UAS-AhR* transgene by *tub-GAL4* and *Act-GAL4* drivers resulted in embryonic lethality. Only a few individuals survived to the larval development stage (Figure 1A). This confirms the existence of endogenous ligands that can affect the human AHR activity in *Drosophila.* Further, the induction of *UAS-AhR* expression by the *Dll-GAL4* driver caused complete lethality of the *Drosophila* pupae, as no adults could hatch. Examination of the leg morphology of the unhatched animals confirmed the complete malformation of the distal leg segments; tarsal segments were missing or severely malformed (Figure 1B–1C).

Interestingly, the effects of the endogenous ligands on the human AHR are limited to a few tissues, as induction of *UAS-AhR* ectopic expression by the *dpp-GAL4* driver (without exogenous ligands) only partially affected wing development (Figure 1H). However, feeding of animals with the exogenous ligands exacerbated the abnormal wing phenotype (Figure 1I) and caused strong leg deformities (Figure 1D–1F). These leg defects were similar to those caused by the ectopic expression of mouse *UAS-AhR* induced by the *dpp-GAL4* driver in *Drosophila* larvae fed with dioxin [31].

**Figure 2:**
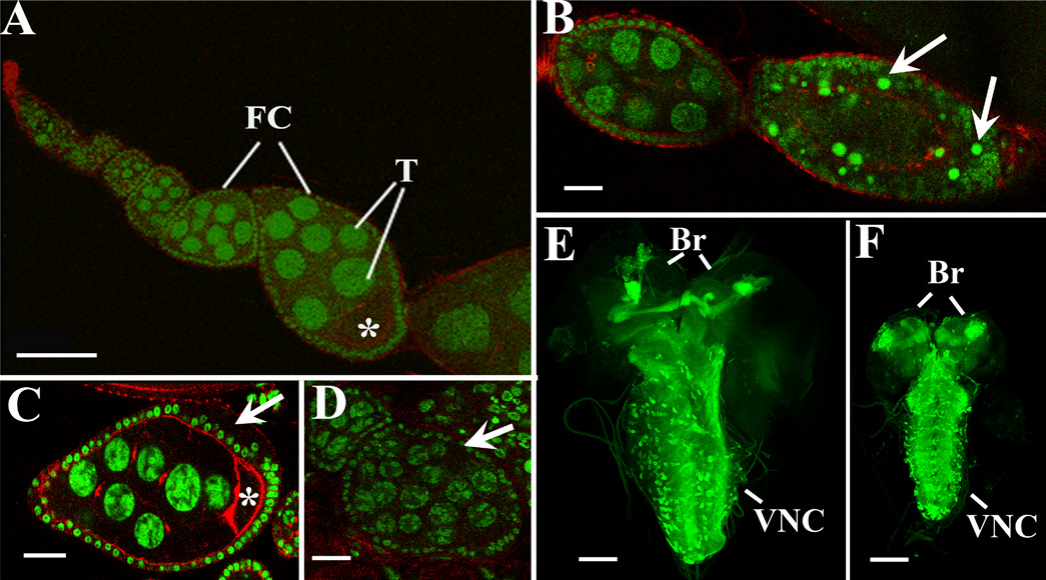
Activation of AHR in germline and nervous systems causes different abnormalities during *Drosophila* oogenesis and neurogenesis (**A**–**D)** Confocal sections of the normal ovariole of *MTD-GAL4/UAS-AhR* female reared on standard medium (A); degraded egg chamber of *MTD-GAL4/UAS-AhR* female fed with beta-Naphthoflavone (arrows point on picnotic nuclei) (B); egg chamber with disordered follicular layer (arrow) from *MTD-GAL4/UAS-AhR* female reared on medium with indinol (C); follicle with 32 trophocytes (arrow) from *MTD-GAL4/UAS-AhR* female fed with indirubin (D). Ovaries were stained with SytoxGreen (green) for DNA visualization and Phalloidin (red) for cytoskeleton visualization. Asterisks, T and FC indicate oocytes, trophocytes, and follicular cells, respectively. (**E**–**F**). Confocal sections of the central nervous system of *UAS-mCD8-GFP*; *UAS-AhR/+*; *Elav-GAL4/+* larvae merged into a single 3D-image. Brains of late third instar larvae developed on standard medium (E) or on the medium containing beta-Naphthoflavone (F). Control brain (E) is significantly bigger than the brain with *AhR* expression (F). *Elav-GAL4* (green, visualized by GFP expression) drives pan-neuronal expression of transgenic *UAS-AhR* in brain hemispheres (Br) and the ventral nerve cord (VNC). Magnification scale bars represent 100 μm in A, E, F and 20 μm in B, C, D.

Induction of *UAS-AhR* expression in the female germ line with an *MTD-gal4* driver, combined with exposure of *MTD-GAL4/UAS-AhR* flies to the exogenous ligands, resulted in a wide range of different abnormalities during oogenesis. The ovary of the wild-type *Drosophila* consists of egg tubes called ovarioles (Figure 2A). The oocyte develops within a group of cells known as an egg chamber (or follicle), which consists of a cluster (or cyst) of 16 germ cells (one oocyte and 15 trophocytes) surrounded by an epithelial monolayer of somatic follicle cells (Figure 2A) [32]. We showed that the ectopic activation of AHR led to the degradation of egg chambers that is evident by the presence of pyknotic nuclei (Figure 2B). The follicular cell layer was often disorganized (Figure 2C). In some instances, we detected cysts with 32 trophocytes suggesting that AHR activation led to an extra round of mitosis during cyst formation (Figure 2D).

Next, we examined *UAS-AhR* expression in the larval nervous system using an *Elav-GAL4* driver. The induction of *UAS-AhR* expression in the absence of the exogenous ligands did not affect the morphology of the *Drosophila* larval nervous system (Figure 2E). However, exposure of the *UAS-AhR/Elav-GAL4* larvae to the exogenous ligands resulted in a smaller brain size and in a shorter ventral nerve cord (Figure 2F), suggesting that the activation of human AHR in tissues of the nervous system could hinder their growth and development.

**Figure 3:**
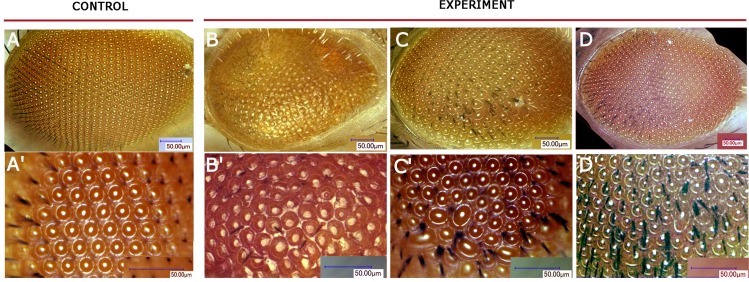
*Drosophila* eye phenotypes of *GMR>AhR* flies Flies developed on standard medium without exogenous ligands (**A**, **A′**), on medium with indinol (**B**, **B′**), beta-Naphthoflavone (**C, C′**) or indirubin (**D, D′**). Ommatidia are arranged in a highly regular pattern in control flies (A–A′), while flies reared on medium with exogenous ligands develop roughened eye phenotypes with irregular pattern and decreased number of mechanoreceptors (B–D, B′–D′).

We also examined eye phenotypes of the flies with induced *UAS*-*AhR* expression in the eye imaginal disks using the *GMR-GAL4* driver. When the *GMR-GAL4/UAS-AhR* flies were raised on standard medium with no exogenous ligands, no defects in eye development were detected: the ommatidia, i.e., the optical units that make up a compound adult fly eye, as well as microchaetae (mechanoreceptors), were packed in a regular array (Figure 3A–3A′). However, the exposure of the *GMR-GAL4*/*UAS-AhR* larvae to the exogenous ligands resulted in a roughened eye phenotype of imagoes (Figure 3B–3B′, Figure 3C–3C′, Figure 3D–3D′). A morphologically similar eye phenotype was observed in flies with dioxin-mediated expression of mouse AHR induced by the *GMR-GAL4* driver [31]. It is worth noting that the magnitude of the eye defects varies depending on the ligand used: *GMR-GAL4/UAS-AhR* flies reared on medium with indinol, demonstrated more severe abnormalities (Figure 3B–3B′) than those reared on medium with indirubin or beta-Naphthoflavone (Figure 3C–3C′, Figure 3D–3D′). Together, these results demonstrate the existence of the endogenous ligand(s) that is/are capable of activating induced human AHR in certain *Drosophila* tissues. However, in other tissues, AHR can be activated only by exogenous ligands making *Drosophila* a valuable model to study the effects of xenobiotics *in vivo,* at an organism level.

### The effects of endogenous and exogenous ligands on the AHR target gene expression in *Drosophila*

To assess the ability of xenobiotics to affect expression of the human AHR target genes in *Drosophila* tissues we carried out a preliminary examination to identify potential human AHR target genes in *Drosophila*. Based on our analysis and on previous studies in humans [33–35] we chose several putative *Drosophila* homologues of the human AHR targets genes. All selected *Drosophila* genes contained *XRE*-elements in their regulatory regions (Supplementary Table 1). This set of putative AHR target genes represents genes that participate in cell proliferation, differentiation, and toxic agent biodegradation (Supplementary Table 2).

**Figure 4:**
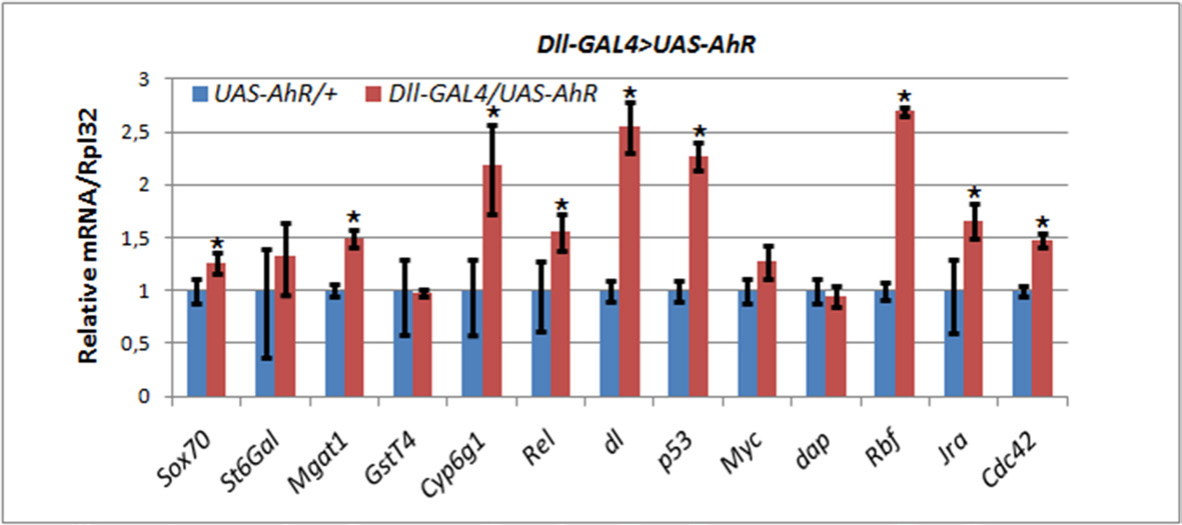
Activation of AHR target genes in leg imaginal discs of *Dll>AhR* larvae in the absence of exogenous ligands mRNA levels in leg imaginal discs of *Dll-GAL4/UAS-AhR* larvae (red) was compared to control *UAS-AhR/+* larvae (blue) developed in the same conditions. The relative level of mRNA expression was measured using real-time PCR. Data are shown as representative of two independent experiments. The error bars represent the measurement error. Asterisk means the significant change in gene expression compared to the control.

The *Dll-GAL4/UAS-AhR* flies raised on the standard medium with no exogenous ligands demonstrated strong leg abnormalities (Figure 1C). Therefore, we chose these animals to examine the effects of endogenous AHR ligands on the expression of AHR target genes. Comparison of the levels of mRNA synthesis by RT-PCR in the leg imaginal discs of the *UAS-AhR* larvae with the *Dll-GAL4/UAS-AhR* larvae developed on the standard medium demonstrated an increase in the transcription levels of most of the examined AHR target genes confirming that the leg imaginal disc tissue may contain endogenous ligand(s) for AHR activation (Figure 4).

To assess the effects of xenobiotics *in vivo* we analyzed expression of the AHR target genes in ovaries of *MTD-GAL4/UAS-AhR* females fed with exogenous ligands for 2 days. In these experiments we used beta-Naphthoflavone, indirubin and indole-3-carbinol (indinol) as the human AHR ligands. We found that, depending on the nature of the exogenous ligand, the induced human AHR had pleiotropic effects on its target genes. Activation of the human AHR by indirubin and beta-Naphthoflavone resulted in the activation of *Cyp6g1* and the suppression of *St6Gal* and *Myc* genes. The activation of the human AHR by indinol resulted in the suppression of *St6Gal* and the activation of *Myc, Cdc42, dl, Mgat1,* and *GstT4* (Table 1, Supplementary Figure 1).

**Table 1:** Summarized results of real-time PCR experiments shown on Supplementary Figures 1–4

Gene Ligand	Indirubin	Beta-Naphthoflavone	Indinol
**Central nervous system of larvae (*Elav-GAL4* driver)**			
*Sox70*	0	+	+
*St6Gal*	+	+	+
*Mgat1*	−	+	+
*GstT4*	−	0	+
*Cyp6g1*	+	+	+
*Rel*	−	+	+
*dl*	+	+	+
*p53*	0	+	+
*Myc*	0	+	+
*dap*	0	+	+
*Rbf*	+	+	+
*Jra*	−	+	+
*Cdc42*	+	+	+
**Adult brains (*Elav-GAL4* driver)**			
*Sox70*	+	+	0
*St6Gal*	+	0	0
*Mgat1*	0	0	0
*GstT4*	+	+	0
*Cyp6g1*	+	+	+
*Rel*	+	+	+
*dl*	0	0	−
*p53*	0	0	−
*Myc*	+	0	0
*dap*	+	0	−
*Rbf*	+	+	+
*Jra*	0	0	−
*Cdc42*	0	0	−
**Ovaries (*MTD-GAL4* driver)**			
*Sox70*	0	+	+
*St6Gal*	0	0	−
*Mgat1*	0	−	−
*GstT4*	+	+	0
*Cyp6g1*	+	+	+
*Rel*	−	+	−
*dl*	0	0	−
*p53*	+	−	0
*Myc*	−	0	−
*dap*	+	+	0
*Rbf*	+	0	−
*Jra*	+	0	0
*Cdc42*	0	0	+
**Eye imaginal discs (*GMR-GAL4* driver)**			
*Sox70*	−	0	+
*St6Gal*	+	0	+
*Mgat1*	−	−	0
*GstT4*	0	0	+
*Cyp6g1*	0	0	0
*Rel*	0	+	+
*dl*	−	0	+
*p53*	0	−	+
*Myc*	−	−	+
*dap*	−	−	0
*Rbf*	0	0	+
*Jra*	0	−	0
*Cdc42*	−	−	0

When human AHR was induced in the central nervous system by the *Elav-GAL4* driver in larvae developed on medium supplemented with indinol or beta-Naphthoflavone, the expression levels of almost all of the target genes significantly increased (Table 1, Supplementary Figure 2). The activation of human AHR by indirubin resulted in an increase in the levels of *St6Gal, Cyp6g1, dl, Cdc42* and *Rbf,* but a decrease in the levels of *Mgat1, GstT4, Rel* and *Jra* genes. However, the induction of human AHR by the *Elav-GAL4* driver in adult brain, combined with feeding with exogenous ligands, generated less pronounced effects on the expression of the AHR target genes. The expression of some genes was not affected by xenobiotics while the expression of other genes reduced (Table 1, Supplementary Figure 3).

The xenobiotic-mediated effect of human AHR activity in eye imaginal disks of *GMR-GAL4/UAS-AhR* larvae also resulted in three modes of the target gene response: an increase in the gene expression, a decrease, and several genes had no response to the AHR activity (Table 1, Supplementary Figure 4).

Together, our results suggest that while the endogenous *Drosophila* ligands of the human AHR mostly led to an increase in the expression of the AHR target genes (Figure 4), the effects of the xenobiotics are pleiotropic depending on the gene and tissue. We detected both increases, decreases and no effects on the expression of multiple target genes in four different tissues: eye imaginal disks, larval central nervous system, adult brain and adult ovaries (Table 1, Supplementary Figures 1–4). The most striking increases in the xenobiotic-driven gene expression were detected in the larval CNS and brain.

### Transcriptional regulation by AHR is mediated by the PcG of epigenetic chromatin modifiers

Upon observation of the striking effects of AHR and its exogenous ligands/xenobiotics on fly development we hypothesized that some AHR target genes may also be under regulation by other developmental regulator networks. In particular, we posed the question whether some AHR target genes are also under the control of other developmental regulators, such as the Polycomb group (PcG) of epigenetic factors, which regulate gene expression by modulating the chromatin structure. This may explain the detected pleiotropic effects of the human AHR ligands during development. To test this hypothesis, we performed experiments using mutant flies with one null-allele of *Polycomb* (*Pc*^4^), a gene which is the key member of the PcG of epigenetic regulators and a component of the PRC1 complex [36–38].

**Figure 5:**
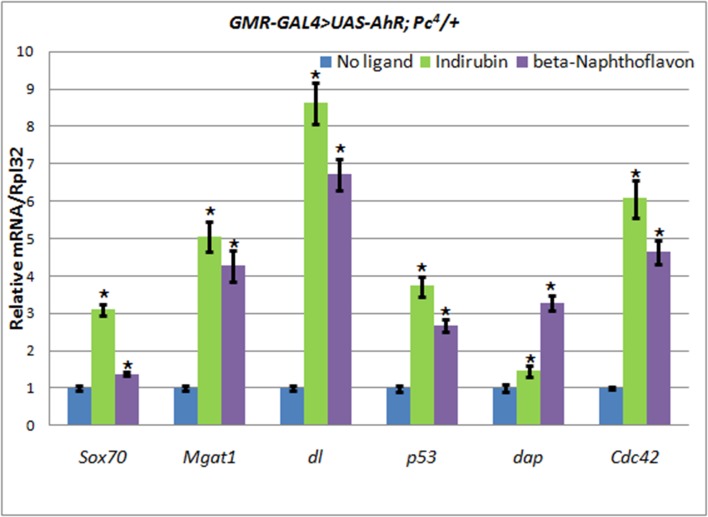
The increase of AHR target genes expression in heads of *GMR-GAL4/UAS-AhR*; *Pc*^4^/+ imagoes Flies developed from larvae grown on medium with added indirubin (green), beta-Naphthoflavone (purple), or standard medium without additives (blue). mRNA levels were analyzed by real-time PCR in heads dissected from *GMR-GAL4/UAS-AhR*; *Pc*^4^/+ imagoes. Data are shown as representative of two independent experiments. The error bars represent the measurement error. Asterisk means the reliable change in gene expression compared to the control.

To examine this hypothesis we compared the levels of expression of the AHR target genes in the heads of wild-type and *Pc*^4^ mutant animals carrying the *UAS-AhR* transgene. To avoid the effect of the endogenous ligands, we induced the expression of the *UAS-AhR* transgene in eye imaginal discs (*GMR-GAL4* driver), and we only chose genes for analysis whose transcription levels in eyes decreased in response to the addition of ligands (beta-Naphthoflavone and indirubin): *Sox70, Mgat1, dl, p53, dap,* and *Cdc42* (Table 1, Supplementary Figure 4B–4C) to the food medium. Indeed, we detected an increase in the transcription levels of the human AHR target genes in *GMR-GAL4/UAS-AhR; Pc*^4^*/+* mutants compared to *GMR-GAL4/UAS-AhR* flies with the wild-type *Pc* gene, both of which were grown on the medium with the exogenous ligands (Figure 5, Table 2).

**Table 2:** The depletion of epigenetic repressors activates AHR target genes expression

Gene symbol	Allele of *Polycomb* and added Inhibitor							
	*Pc*^+^		*Pc*^−^		*Pc*^+^ + UNC1999		*Pc*^+^ + Belinostat	
	Ligand							
	Indirubin	BNF	Indirubin	BNF	Indirubin	BNF	Indirubin	BNF
*Sox70*	–	0	+	+	+	+	+	+
*Mgat1*	–	–	+	+	+	+	+	+
*dl*	–	0	+	+	+	+	+	+
*p53*	0	–	+	+	+	+	+	+
*dap*	–	–	+	+	+	+	+	+
*Cdc42*	–	–	+	+	+	+	+	+

**Figure 6:**
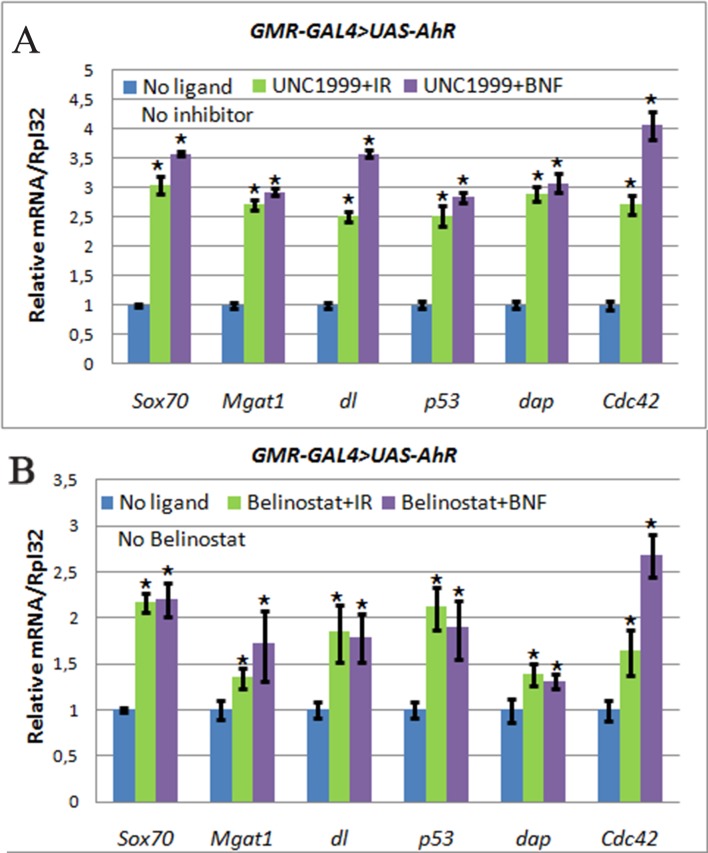
The increase of AHR target genes expression in heads of *GMR>AhR* imagoes Flies developed from larvae grown on medium with UNC1999 (**A**), Belinostat (**B**), and ligands, indirubin (IR, green) and beta-Naphthoflavone (BNF, purple), or standard medium without additives (blue). mRNA levels were analyzed by real-time PCR in heads dissected from *GMR-GAL4/UAS-AhR* imagoes. Data are shown as representative of two independent experiments. The error bars represent the measurement error. Asterisk means the reliable change in gene expression compared to the control.

Summarized results of real-time PCR experiments shown on Supplementary Figures 4–6. “*Pc*^+^” column represents the results for selected genes from Supplementary Figure 4 with decreased or maintained mRNA expression. “*Pc*^−^”, “*Pc*^+^ + UNC1999” and “*Pc*^+^ + Belinostat” columns represent the results shown on Figure 5 (for “*Pc*^−^”) and Figure 6. «+», «–» and «0» mean the increasing expression, the decreasing expression and no effect, respectively. BNF, beta-Naphthoflavone.

Given the importance of chromatin modifiers in cancer and other disease [39–41], significant efforts are being made to develop small molecule inhibitors of these enzymes (epigenetic inhibitors) and some are already available. To confirm the above results for *Pc* mutant flies and to examine potential cumulative effects of epigenetic inhibitors and xenobiotics, we examined the effect of UNC1999, a specific inhibitor of another key member of the PcG family, E(z), the only H3K27me3 histone methyltrasferase and a member of the PRC2 complex, in flies [42]. In addition, we used belinostat, an inhibitor of histone deacetylases (HDACs). Both inhibitors led to a similar general decondensation of the chromatin structure and, as a result, gene activation. Interestingly, the using of both inhibitors led to the similar, albeit less robust, increase in the expression of the AHR target genes in the presence of both xenobiotics (Figure 6, Table 2). The results of these experiments suggest that in some tissues and during certain periods of *Drosophila* development many AHR target genes are at the same time regulated by the chromatin-based epigenetic mechanisms of transcriptional regulation.

## DISCUSSION

### Endogenous ligands activate human AHR in the *Drosophila* leg and wing imaginal discs and during embryogenesis

Our phenotypic analysis revealed that ectopic expression of human AHR in various *Drosophila* organs and tissues affected their development. Interestingly, in some tissues, the effect was detected in the absence of exogenous ligands, while in other tissues it occurred only in the presence of exogenous ligands/xenobiotics. For example, the induction of ectopic expression of *UAS-AhR* by the *tub-GAL4, Act-GAL4, dpp-GAL4* and *Dll-GAL4* drivers, caused phenotypic effects in the absence of the exogenous AHR ligands. This suggests that endogenous ligands can activate the human AHR in the *Drosophila* embryo, as well as in the wing and leg imaginal discs. Further molecular analysis of the expression of the AHR target genes in the leg imaginal discs confirmed our conclusion: the transcription levels of the majority of these genes increased despite the absence of exogenous ligands capable of activating of AHR (Figure 4). Since *Dll-GAL4* drives *UAS-AhR* expression in the tarsal cap and the proximal ring of the leg imaginal discs in a very small number of cells comparative to the remaining bulk of the disc cells [43], the observed increase in the transcription levels of the AHR target genes is likely to be quite high. This ability of the human AHR to respond to the endogenous invertebrate ligands indicates a conservation of the structural and functional features of the *AhR* gene during the evolution of eukaryotes.

It is important to note that both the *Dll-GAL4* and *dpp-GAL4* drivers activate *UAS*-mediated human AHR expression in the leg imaginal discs, but the phenotypic effects of human AHR activation by endogenous ligands (defective leg segmentation) were observed only in the *UAS-AhR/+;Dll-GAL4*/+ flies (Figure 1C). Since *Dll-GAL4* and *dpp-GAL4* drivers have different expression patterns within the leg imaginal discs, this indicates that the effect of the endogenous ligands on AHR activity is not only tissue, but also cell-type specific. Accordingly, in tissues with no *Drosophila* endogenous ligands capable of activating the human AHR, its activation requires exogenous xenobiotics.

### Using *Drosophila* as a model to assess function of the human AHR

To activate human AHR by exogenous ligands, we used only molecules which are known to act as agonists of this receptor, i.e. cause only an increase in the transcription levels of AHR target genes in mammals [35]. Unexpectedly, in addition to an increase, we found a decrease and also unchanged expression levels of the *Drosophila* AHR target genes. The range of changes in the levels of transcription was very wide and time and tissue-specific for each of the ligands used (Table 1). We found increases in the transcription levels from several percentage points in fully differentiated tissues up to several hundred folds in tissues with actively proliferating cells. The decrease in transcription was from a few percent to almost complete suppression (Supplementary Figures 1–4). We concluded that the human AHR may have tissue- and cell-specific effects on its target genes and that these effects are diverse and depend on the nature of the ligands, target genes and on the developmental stage. Importantly, our work is the first to show that *Drosophila* may serve as an important model organism to assess complex pleitropic effects of xenobiotics on the activity of the human AHR.

### Transcriptional regulation by AHR is linked to developmental activities of epigenetic modulators of chromatin structure

It is possible that our inability to detect expected increases in the expression of some AHR target genes in particular tissues may reflect the tight chromatin architecture of the target gene regulatory regions. This tight chromatin may impede the accessibility of the DNA binding sites to the AHR/ARNT transcription complex. The condensed structure of nucleosomes is usually associated with particular modifications of nucleosomal histones, such as a lack of acetylated residues and the presence of methylated residues, particularly in H3K27me3. These condensed, repressive chromatin structures are the products of the activities of the HDACs and PcG complexes, PRC1 (containing Pc) and PRC2 (containing histone methyltrasferase E(z)). In concert, these protein complexes are involved in epigenetic gene silencing by condensing the structure of chromatin [44–48].

Indeed, we confirmed this hypothesis by showing that genetic depletion of *Pc*, inhibition of HDACs by belinostat, and inhibition of E(z) H3K27me3 activity by UNC1999 leads to an increase in the expression of some AHR target genes using ectopic expression of the human AHR and some of its exogeneous ligands (Figures 5–6, Table 2).

The importance of this study is that it presents *Drosophila* as a valid model organism to study the *in vivo* effects of xenobiotics, i.e. the ligands of the human AHR during development. This is facilitated by the limited effects of the endogenous ligands in this model organism. An additional advantage of this model is that it provides the ability to study the input of the epigenetic modifiers in the functioning of the human AHR in the context of the organism, where these factors were discovered and best studied during development. In recent years, the effect of the exogenous AHR ligands on different types of cancer has been intensively studied. As a result, new anticancer agents were discovered and set into production [49, 50]. However, their effect is not selective and strongly depends on the type of tumor [49]. At the same time, many pharmaceutical companies are rapidly developing small molecule inhibitors of histone-modifying enzymes, such as UNC1999 or HDAC inhibitors, with the hope that they can also be used as anti-cancer drugs [51, 52]. Importantly, our studies provide the first result that may merge both approaches *in vivo*, in that we found that inhibition of the activities of the *PcG* genes can modulate the action of the AHR exogenous ligands. Our results imply that in treating disease conditions caused by xenobiotics, it is essential to consider the applications of different types of pharmaceutical agents, not only affecting the effects of xenobiotics, but also affecting enzymes which modulate transcription of common target genes through changes in chromatin structure.

## MATERIALS AND METHODS

### Fly Stocks, Rearing Conditions and Reagents

*UAS-AhR* strain with inducible human *AhR* gene expression was obtained in this study as described below. Wild type *Oregon R, w*^*1118*^ and *GAL4-*driver lines were obtained from the Bloomington *Drosophila* stock center. The following *GAL4* lines were used to drive the expression of the *UAS-AhR* construct: for ubiquitous expression - *tub-GAL4* (genotype: *y*^*1*^w^*^; P{tubP-GAL4}LL7/TM3,Sb^*1*^*Ser*^*1*^) and *Act-GAL4* (genotype: *y*^*1*^w^*^; P{Act5C-GAL4-w}E1/CyO); for expression in leg and wing imaginal disks - *dpp-GAL4* (genotype: *y*^*1*^*w*^*67c23*^*; P{dpp-GAL4.PS}6A/TM6,Tb*^*1*^) and *Dll-GAL4* (genotype: *P{GawB}Dllmd23/CyO*); in eye imaginal disks - *GMR-GAL4* (genotype: w^*^; P{GAL4-ninaE.GMR}12); in the central nervous systems - *Elav-GAL4* (genotype: *P{GawB}elav*^*C155*^*, P{UAS-mCD8::GFP.L}Ptp4E*^*LL4*^, P{hsFLP}1, w^*^/FM7c), in germ line cells - *MTD-GAL4 (*genotype: P{otu-GAL4::VP16.R}1, w^*^; *P{GAL4-nos.NGT}40; P{GAL4::VP16-nos.UTR}CG6325^MVD1^*). *Pc*^*4*^*p*^*1*^*e*^*5*^*/TM6C* and *UAS-GFP* lines are the gift from Dr. Maxim Erokhin (Institute of Gene Biology Russian Academy of Science).

In genetic experiments we used standard Formula 4-24 medium (Carolina Biological Supply, USA). Following ligands were used: 2′Z-Indirubin (SML0280, Sigma-Aldrich, USA), beta-Naphthoflavone (A18543, Alfa Aesar, Thermo Fisher Scientific, UK), Indole-3-Carbinol (indinol, Mirax Biopharma, Russia). Following inhibitors were used: UNC1999 (SML0778, Sigma-Aldrich, USA), belinostat (PXD101, Sigma-Aldrich, USA). Ligands and inhibitors were diluted in solution according to the manufacturer’s protocol and added to the Formula 4-24 medium at a corresponding final concentration: beta-Naphthoflavone - 200 mkg/g medium, indirubin - 25 mkg/g medium, indole-3-carbinol - 10 mg/g medium, UNC1999 - 20 mkg/g medium, belinostat - 20 mkg/g medium.

Ligands and inhibitors were fed to imago or larval offspring obtained after crossing the *GAL4-*driver flies with *UAS-AhR* flies. Parents were kept on standard Formula 4-24 medium, and then the larval offspring of late 2nd stage was selected for feeding experiments. Larvae and flies were kept at room temperature (25°C).

To obtain flies with *GMR-GAL4/UAS-AhR; Pc*^*4*^/+ genotype we crossed *UAS-AhR; Pc*^*4*^*p*^*1*^*e*^*5*^*/TM6C* females with *GMR-GAL4/Cy0* males and flies without balancer chromosomes were further selected in the offspring.

### Generation of transgenic *UAS-AhR* flies

Homo sapiens *AhR* cDNA was taken from pCMV6-XL4 construct obtained from OriGene Technologies, Inc., clone SC119159 (NM_001621.2). A 5 kb human cDNA was cloned into the Not I restriction site in the *pUAST* vector. The correct orientation of the insert was proven by sequencing. The resulting *UAS-AhR* plasmid construct was injected into the early-stage (*w*^*1118*^) embryos using standard technique for the P-element dependent transformation [53], and *w*^+^-positive transformants were selected by standard genetic methods to select *Drosophila UAS-AhR* transgenic line. The site of the *UAS-AhR* insertion was genetically mapped on the 2nd chromosome.

The presence of the human *UAS-AhR* construct in *Drosophila* genome was confirmed by PCR using a pair of Ahr1f and Ahr1rev primers (Supplementary Figure 5A). Inverse PCR analysis revealed that only one copy of *UAS-AhR* was inserted in 60E11 cytological region between *CG30424* and *Rpl19 loci* (40 bp upstream of unknown *CG30424* and approximately 540 bp downstream of *Rpl19*). Proper *UAS*-mediated inducible expression of the human *UAS*-*AhR* transgene was confirmed by RT-PCR and Western blot analysis of progeny obtained from crossing of *UAS-AhR* and *Elav-GAL4* flies, grown on the standard medium and the medium with indinol (Supplementary Figure 5B–5C). PCR, RT-PCR and Western blot analysis were performed according to standard protocols [54]. *Tubulin* was used as the control for normalization. Polyclonal rabbit antibody Anti-AHR was used in dilution 1:1000 (PA5-29642, ThermoFisherScientific, USA). Mouse antibody Anti-Actin clone C4 was used in dilution 1:1000 (MAB1501, Merck Millipore, USA).

### Real-time reverse-transcription PCR analysis

Flies/larvae/organs were frozen in liquid nitrogen and total RNA was extracted using RNAzol RT reagent (Sigma-Aldrich, USA) according to the manufacturer’s specifications. RNA Samples were treated with DNase (Turbo DNA-free kit, Applied BioSystems, Life Technologies, USA) according to the manufacturer’s protocol to remove genomic DNA contamination. cDNA was synthesized from 1–5 µg of total RNA, using a cDNA synthesis kit with oligo-dT priming (Thermo Fisher Scientific, USA). The levels of mRNA expression were measured with Real-Time Quantitative Reverse Transcription PCR using TaqMan^®^ probes (Syntol, Russia). All reactions were carried out in triplicate. Real-time PCR was conducted using an ABI Prism 7500 Sequence Detection System (Applied BioSystems, Life Technologies, USA). The 2-ΔΔCt method was chosen as the calculation method [55]. The difference in the cycle threshold (Ct) value of the target gene and its housekeeping gene (*Rpl32*) called ΔCt was calculated using the following equation: ΔΔCt = (ΔCt of ligand treated flies) – (ΔCt of the untreated control flies). Sequences of primer pairs and TaqMan^®^ probes are summarized in Supplementary Table 3.

### Immunohistochemistry

Ovaries were dissected in PBS and fixed for 15 min in 4% paraformaldehyde in PBS + 0.2% Triton X-100. Following fixation, samples were rinsed 3 times with PBS + 0.2% Triton X-100, incubated for 1 hr in CF594 Phalloidin (Biotium, 1:40 dilution), then rinsed 3 times in PBS. Ovaries were incubated for 15 min in RNase A (100 mg/ml), and rinsed again 3 times with PBS and incubated for 15 min in SytoxGreen (Thermo Fisher Scientific, 1:500 dilution), washed with PBS, and mounted in Vectashield mounting medium (H-1000, Vector laboratories).

For preparation and examination of larval CNS, the 3rd instar larvae were immediately washed with ice cold PBS and kept on ice until dissection. Dissected larval CNS were fixed in 4% paraformaldehyde in PBS for 20 min, washed three times in PBS and mounted in Vectashield mounting medium (H-1000, Vector laboratories). GFP was visualized without staining.

### Microscopic analysis

Cuticle preparations of legs and wings were made as described previously [56] and examined using Olympus AH-2 Vanox light microscope. Eyes were scanned with Keyence VHX-1000E digital microscope. Preparations of ovaries and larval CNS were examined using Leica TCS SP5 confocal microscope.

## SUPPLEMENTARY MATERIALS FIGURES AND TABLES



## Abbreviations

AHR: Aryl Hydrocarbon Receptor
PcG: Polycomb Group
PRC: Polycomb Repressive Complex
E(z): Enhancer of zeste
DACs: Histone Deacetylases
GFP: Green Florescent Protein
UAS: Upstream Activation Sequence
