# Supplementary material for: Xenobiotic-induced activation of human aryl hydrocarbon receptor target genes in Drosophila is mediated by the epigenetic chromatin modifiers
Source: Oncotarget. 2017 Oct 31;8(61):102934–47. doi: 10.18632/oncotarget.22173 (PMC5732701; doi:10.18632/oncotarget.22173)
Supplement: Supplementary file 1 [file oncotarget-08-102934-s001.pdf]

Xenobiotic-induced activation of human aryl hydrocarbon receptor target genes in *Drosophila* is mediated by the epigenetic chromatin modifiers

SUPPLEMENTARY MATERIALS

Supplementary Table 1: Positions of putative *XREs* within selected *Drosophila* gene's sequences

Gene symbol	Putative *XRE*	Position relative to TSS	mRNA accession ID
GstT4	TCTTTGTGCCAACTGTTGTGCCACCTTGT<br>GCGGAGCTCAGTCTAGCAGCAAAAGTCC	1227 bp upstream<br>2 bp downstream	NM_132658.2
Cyp6g1	AGTTTCCTTGAAATTGCTGGACAAACTT<br>AGATTTTCTATTTAGCTTTACAAGCAG	73 bp downstream<br>436 bp downstream	NM_001299406.1
dap	ATGAGCAGAAAACTTGCGTTTCACAAAG<br>CTGACCTCCTCCTGGGGTGGAATGGGCT<br>ATGAGTTGCCATATAGAGTGGGATATCT	1006 bp upstream<br>2059 bp upstream<br>2086 bp upstream	NM_057600.6
Myc	CAAAAGCTCGCAAATTGCGGGACCATTT<br>TGAACCCTCGACATCGCCTGACGTCATT	1825 bp upstream<br>1552 bp downstream	NM_001272275.1
dl	TCGACGTCACGCGCTCTCTCGCGCT	547 bp downstream	NM_165218.3
Jra	CGAATGTTCCGAGTTAAGTGTCATATTT<br>CTAAGCTTCTTGCTTGAGTCAATGCTGC	445 bp upstream<br>62 bp upstream	NM_057238.5
Mgat1	GAACAAGTCAATCGAGCTTCCAGTGCAG	152 bp downstream	NM_080378.4
p53	AGAAATTTCACCATACGCCGATGCGCGT<br>ACCCAACTGATGCCATCTAGAGGACCTG<br>TTGTAACTCGAGCAAGCTGGAACATGAA	482 bp upstream<br>361 bp upstream<br>7 bp upstream	NM_001260323.2
Rbf	GTGGATCACACGCACCTGCCCGCCGCAA<br>GCGCCAGGCCCGCAACGCCCCGGTTAGC	158 bp downstream<br>550 bp downstream	NM_080297.3
Rel	GCGTTTTGCTCGCAACTCGTGAGCGATA	342 bp upstream	NM_206466.1
St6Gal	GGGAATTTCTCGATAATTGGGAATAAAAT	421 bp downstream	NM_166684.2
Sox70	TATGCTCTTACGCTATGGGCCAGGTATA	404 bp upstream	NM_079342.3
Cdc42	AGAAAGCTCAAAGTGGTTTGAGCAGAAGT<br>TCCGTGCCGCAGTGTGCGTGTGTGATAA	701 bp upstream<br>283 bp downstream	NM_001258833.3
Rpl32	–	–	NM_170461.3
spire	–	–	NM_001299171.1

Supplementary Table contains coordinates of putative *XRE* consensus within selected *Drosophila* gene's sequences relative to TSS (Transcription Start Site). For predicting of human AHR binding sites in *Drosophila* genome we used matrix (TRANSFAC Accession Number M00139) from Transfac database implemented in LASAGNA-Search 2.0 web tool with default parameters for all genes [57]. Only significant hits (p-value ≤ 0.001) were retained. It should be mentioned that we were not able to detect any *XRE* in *Rpl32* (reference gene in qPCR experiments) and *spire* (randomly chosen gene) using the same parameters of search. This indicates that not every randomly chosen gene contains putative *XRE*.

Supplementary Table 2: The selected set of the AHR target genes in Drosophila used in the RT-PCR analysis

Gene symbol	Gene name	Biological process
Cyp6g1	*Cyp6g1*	Oxidation-reduction process, response to DDT, insecticide catabolic process, response to insecticide, sleep.
dap	*dacapo*	Cell cycle arrest, negative regulation of the cell cycle, regulation of the cyclin-dependent protein serine/threonine kinase activity.
Cdc42	*Cdc42*	Actin cytoskeleton organization, actin filament polymerization, vesicle-mediated transport, dendrite development, regulation of wound healing, regulation of carbohydrate metabolic process, cellular response to stress.
Myc	*Myc*	Transcription factor, homologous to the vertebrate Myc proto-oncogene, cell cycle, regulation of cell size, cell proliferation, regulation of carbohydrate metabolic process, positive regulation of peptide secretion, response to stress, programmed cell death, regulation of Notch signaling pathway.
dl	*Dorsal*	Transcription factor activity, RNA polymerase II distal enhancer sequence-specific binding, Toll signaling pathway, positive regulation of immune system process, dorsal/ventral axis specification.
GstT4	*Glutathione S transferase T4*	Glutathione metabolic process, biological oxidations
Jra	*Jun-relatedantigen*	Positive regulation of metabolic process, humoral immune response, multicellular organism aging, regulation of response to external stimulus, synaptic growth at neuromuscular junction, RNA polymerase II transcription factor activity.
Mgat1	*Mannosyl (α-1,3-)-glycoprotein β-1,2-N-acetylglucosaminyl transferase 1*	Acetylglucosaminyltransferase activity, determination of adult lifespan, adult locomotor behavior, mushroom body development, protein N-linked glycosylation.
p53	*p53*	Ubiquitin protein ligase binding, transcription factor activity, TFIIH-class transcription factor binding, response to radiation, regulation of response to stress, negative regulation of growth, aging, regulation of apoptosis involved in tissue homeostasis, germ cell development.
Rbf	*Retinoblastoma-family protein*	RNA polymerase II regulatory region DNA binding, negative regulation of the cell cycle G2/M phase transition, negative regulation of G1/S transition of mitotic cell cycle, response to extracellular stimulus; regulation of nucleic acid-templated transcription, apoptotic signaling pathway.
Rel	*Relish*	RNA polymerase II transcription factor activity, positive regulation of gene expression, cell projection organization, response to starvation, RNA biosynthetic process, negative regulation of stem cell proliferation, sensory perception, neuron projection morphogenesis.
Sox70	*Dichaete*	Positive regulation of nucleobase-containing compound metabolic process, intracellular mRNA localization involved in pattern specification process, neuroblast development, dorsal/ventral pattern formation, dorsal/ventral axis specification, sequence-specific DNA binding, transcription factor activity, RNA polymerase II distal enhancer sequence-specific binding.
ST6Gal	*Sialyl transferase*	Sialyltransferase activity, protein glycosylation, neuromuscular junction development, oligosaccharide metabolic process.

Supplementary Table 3: Sequences of primer pairs and TaqMan® probes used in this study

Gene symbol	Title	Sequences (5′–3′)
Sox70	dsox70f	TTCCATCAACTACCGCCATA
	dsox70r	GTACGGCACTGGATAACCCT
	dsox70taq	FAM-TGCTGTTCTCGTCGCGCAAA-BHQ1
ST6Gal	st6f	CACAACAAGACCCAGCAGTT
	st6r	GTCCACGTTGTAGTGGTTCG
	st6taq	FAM-CCTTCTCGAAGGCCCGGACA-BHQ1
Mgat1	mgat1f	TGATTTCAAGAGCGGTGTTC
	mgat1r	GGCGGTACTCTGTCCTTAGC
	mgataq	FAM-TACAACAAACGGCGCGTGCA-BHQ1
GstT4	cg1681f	TTCGCACCCACTCTAGTCAC
	cg1681r	GCTCGATTGGTTCAGGAAAT
	cg1681taq	FAM- TCAACGAGATGTCGCAGCCACTC-
Cyp6g1	Cyp6g1f	GCGATCCATTGGGCTATAAT
	Cyp6g1r	CCAATCTCCTGCATAAGGGT
	Cyp6g1taq	FAM- TCGCACCAAGCTGACTCCCG-BHQ1
Rel	Relf	GAAAGTAGCGATGCTGGTCA
	Relr	TGTTGTCCATTTCGGTGTCT
	Reltaq	FAM-TCCAACTCCACGGAATCCTCGTC-BHQ
dl	Dlf	CTCCTGCGTCACAAAGGATA
	Dlr	GCTCGCATTGTCTCACTGTT
	Dltaq	FAM-TCTTGCAGCCCTCCTTGCCA-BHQ1
p53	p53f	GTACTCGATTCCGCTGAACA
	p53r	CACGCAAATTAAGTGGTTGG
	p53taq	FAM-CTGAACGTCCACGTTGAAGGCC-BHQ
Myc	dmf	CCGCGCTACAATAACTTCAA
	dmr	GCAGTTCTGATACGGTGTGC
	dmtaq	FAM-TCGGTGGCCAACTCGCGTTA-BHQ1
dap	dapf	CAGAGATGTACACCCTAA
	dapr	GGAGTCGTAACAAGATTC
	daptaq	FAM-TTATCCGTGTTCGACTCTAGCG-BHQ1
Jra	jraf	TTCACACTAACTCCCAGGCA
	jrar	CTCGGTCATGTTGGTGTAGG
	jrataq	FAM-CAACTGCGGCAGCCATGACA-BHQ
Rbf	Rbf	CTGGCGAAGAGGTAATAGCC
	Rbr	GGACTTCGCTAGTTGGAAGC
	Rbtaq	FAM-CTTCGCCTCCGTTGACGGGT-BHQ1
Cdc42	cdc42f	CGAGATTACACACCATTGCC
	cdc42r	ATGGGCTTCTGCTTGTTCTT
	cdc42taq	FAM-TGCTGTTCTCGTCGCGCAAA-BHQ1
Rpl32	Rpl32dir	CCAGCATACAGGCCCAAGATC
	Rpl32rev	ACGCACTCTGTTGTCGATACC
	Rpl32probe	FAM-CGCACCAAGCACTTCATCCGCCAC-BHQ1
AhR	Ahr1f	TGCTGCCTTTCCCACAAGAT
	Ahr1rev	CTACTGTCTGGGGGAGACCA
Tubulin	Tub1f	TTGTCGCGTGTGAAACACTTC
	Tub2rev	ACGTCCTTGGGCACAACATC

FAM fluorescent dye (6-carboxy fluorescein) was used for labeling oligonucleotides. BHQ1 (Black Hole Quencher-1) is used as a dark quencher.

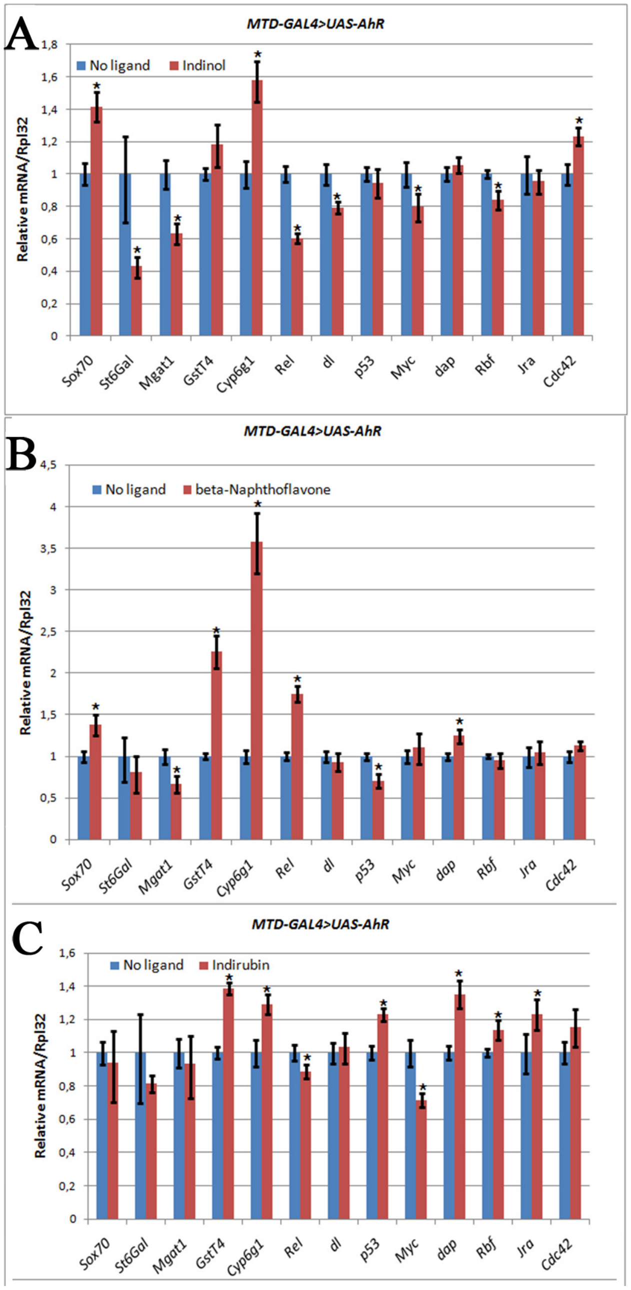

Supplementary Figure 1: Changes in expression of AHR target genes in ovaries of *MTD>AhR* females. Flies were reared on medium with indinol (**A**), beta-Naphthoflavone (**B**), or indirubin (**C**) (red), or without additives (control, blue) for 2 days. mRNA levels were analyzed by real-time PCR in ovaries dissected from *MTD-GAL4/UAS-AhR* females. Data are shown as representative of two independent experiments. The error bars represent the measurement error. Asterisk means the reliable change in gene expression compared to the control.

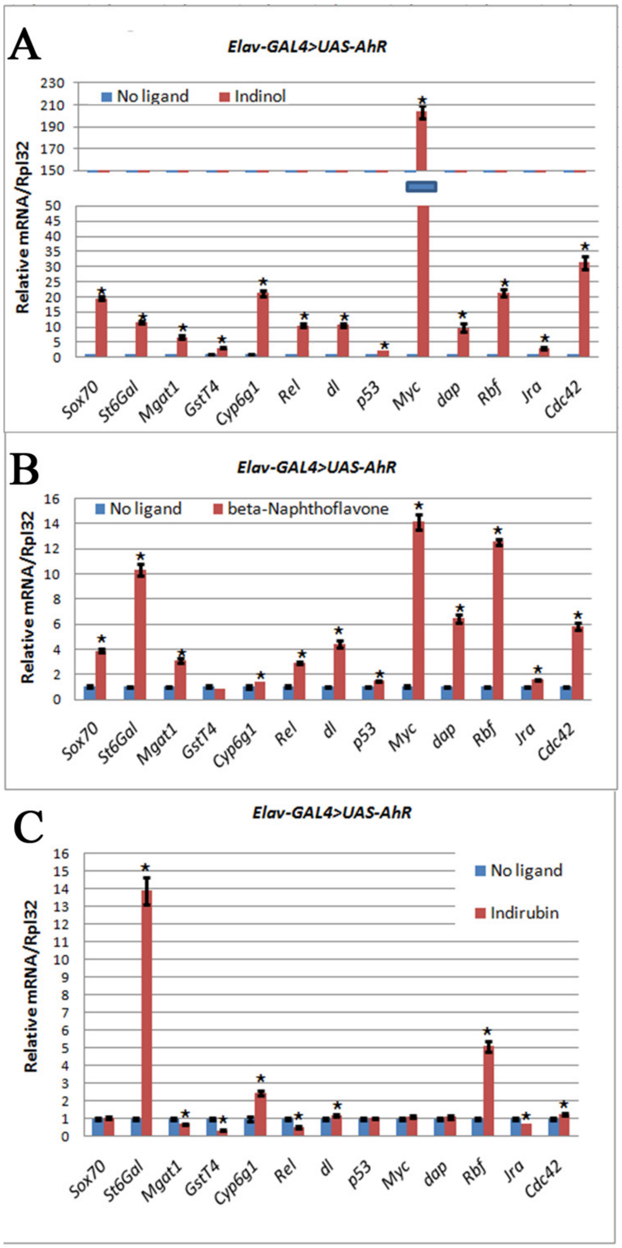

Supplementary Figure 2: Changes in expression of AHR target genes in the central nerve system of *Elav>AhR* larvae. Larvae were reared on medium with indinol (**A**), beta-Naphthoflavone (**B**) or indirubin (**C**) (red), or without additives (control, blue). mRNA levels were analyzed by real-time PCR in CNS dissected from *Elav-GAL4/UAS-AhR* larvae. Data are shown as representative of two independent experiments. The error bars represent the measurement error. Asterisk means the reliable change in gene expression compared to the control.

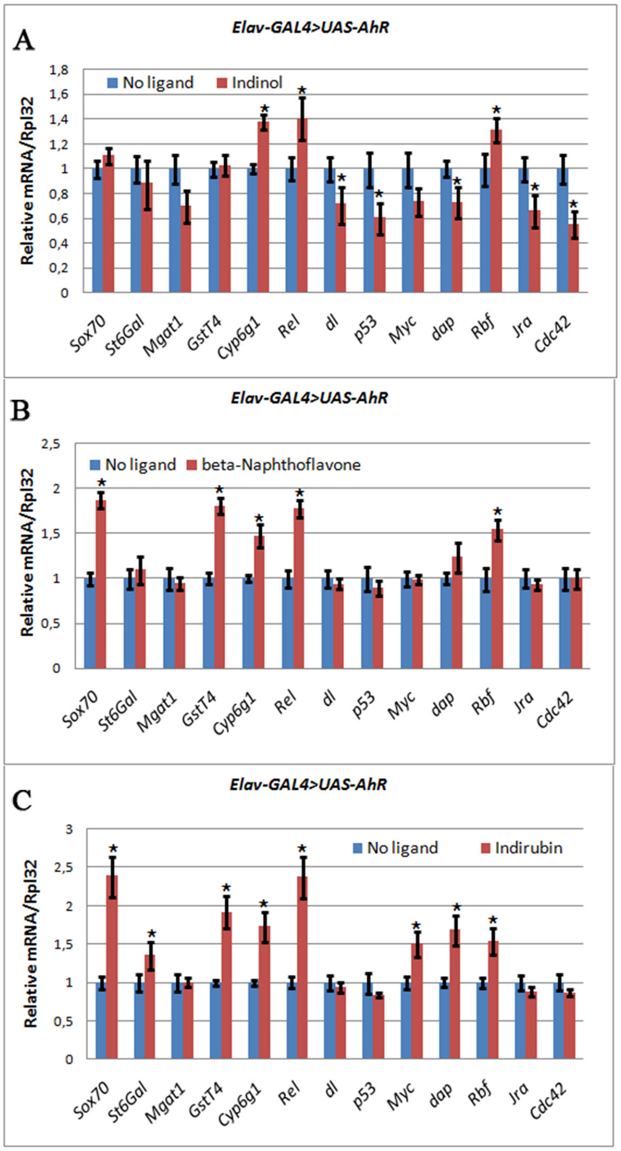

Supplementary Figure 3: Changes in expression of AHR target genes in heads of *Elav>AhR* imagoes. Flies developed from larvae grown on medium with added indinol (**A**), beta-Naphthoflavone (**B**) and indirubin (**C**) (red), or without additives (blue). mRNA levels were analyzed by real-time PCR in heads dissected from *Elav-GAL4/UAS-AhR* adult flies. Data are shown as representative of two independent experiments. The error bars represent the measurement error. Asterisk means the reliable change in gene expression compared to the control.

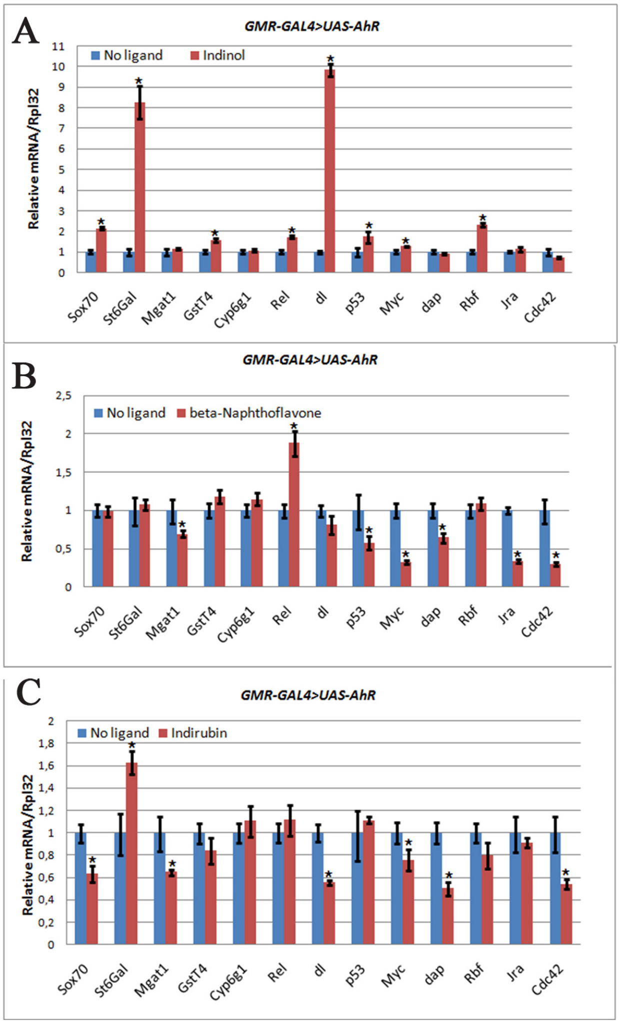

Supplementary Figure 4: Changes in expression of AHR target genes in heads of *GMR>AhR* imagoes. Flies developed from larvae grown on medium with added indinol (**A**), beta-Naphthoflavone (**B**) and indirubin (**C**) (red), or without additives (blue). mRNA levels were analyzed by real-time PCR in heads dissected from *GMR-GAL4/UAS-AhR* adult flies. Data are shown as representative of two independent experiments. The error bars represent the measurement error. Asterisk means the reliable change in gene expression compared to the control.

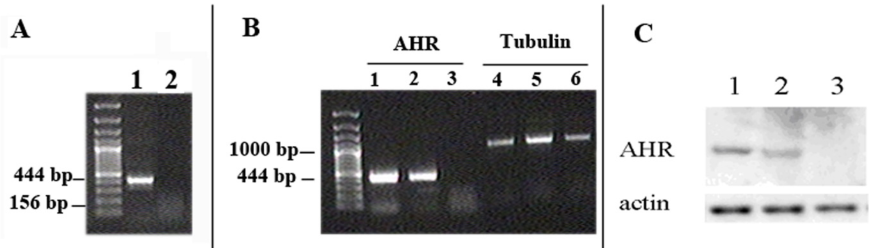

Supplementary Figure 5: Analysis of *AhR* transgenic expression. (**A**) PCR analysis of genomic DNA using *AhR*-specific primers. 1, *UAS-AhR* flies. 2, w^{1118} flies (control). (**B**) RT–PCR of mRNA from heads of *Elav-GAL4/+, UAS-AhR/+* flies grown on medium with indinol (1, 4) or without additives (2, 5), and from heads of *UAS-AhR* flies grown on medium without additives (3, 6). RT-PCR was used with specific primers to detect the expression of *AhR* (1–3) or *tubulin* as loading control (4–6). (**C**) Western blot analysis of AHR expression in heads of *Elav>hAhR* flies grown on medium with indinol (1) or standard medium without exogenous ligands (2), and in heads of *UAS-AhR* flies as negative control (3). Actin was used as loading control.
